# Comparison of TIVA and Desflurane Added to a Subanaesthetic Dose of Propofol in Patients Undergoing Coronary Artery Bypass Surgery: Evaluation of Haemodynamic and Stress Hormone Changes

**DOI:** 10.1155/2016/3272530

**Published:** 2016-07-27

**Authors:** Didem Onk, Tülin Akarsu Ayazoğlu, Oruç Alper Onk, Mehmet Aksüt, Murat Günay, Kultigin Turkmen, Aynur Özensoy, Çiğdem Yazıcı Ersoy, Abdulkadir Çoban

**Affiliations:** ^1^Department of Anesthesiology, Erzincan University, 24030 Erzincan, Turkey; ^2^Department of Anesthesiology, Göztepe Training and Research Hospital, 34722 Istanbul, Turkey; ^3^Department of Cardiovascular Surgery, Erzincan University, 24030 Erzincan, Turkey; ^4^Department of Cardiovascular Surgery, Kartal Koşuyolu High Specialization Training and Research Hospital, 34865 Istanbul, Turkey; ^5^Department of Biochemistry, Erzincan University, 24030 Erzincan, Turkey; ^6^Department of Internal Medicine, Division of Nephrology, Necmettin Erbakan University, Meram School of Medicine, 42080 Konya, Turkey; ^7^Department of Anesthesiology, Kartal Koşuyolu High Specialization Training and Research Hospital, 34865 Istanbul, Turkey; ^8^Department of Internal Medicine, Şişli Etfal Training and Research Hospital, 34363 Istanbul, Turkey

## Abstract

*Introduction*. Increased levels of stress hormones are associated with mortality in patients undergoing coronary artery bypass grafting (CABG).* Aim*. To compare total intravenous anaesthesia (TIVA) and desflurane added to a subanaesthetic dose of propofol.* Material and Methods*. Fifty patients were enrolled in this study. Fentanyl (3–5 mcg/kg/h) was started in both groups. Patients were divided into two groups. The PD group (*n* = 25) received 1 minimum alveolar concentration (MAC) desflurane anaesthesia in addition to propofol infusion (2-3 mg/kg/h), while P group (*n* = 25) received propofol infusion (5-6 mg/kg/h) only. Biochemical data, cortisol, and insulin levels were measured preoperatively (T0), after initiation of CPB but before cross-clamping the aorta (T1), after removal of the cross-clamp (T2), and at the 24th postoperative hour (T3).* Results*. Systolic, diastolic, and mean arterial pressure levels were significantly higher in PD group than those in P group in T1 and T2 measurements (*p* ≤ 0.05). CK-MB showed a significant decrease in group P (*p* ≤ 0.05). When we compared both groups, cortisol levels were significantly higher in PD group than P group (*p* ≤ 0.05).* Conclusion*. Stress and haemodynamic responses were better controlled using TIVA than desflurane inhalation added to a subanaesthetic dose of propofol in patients undergoing CABG.

## 1. Introduction

A stress response is defined as the hormonal and metabolic changes that occur following injury and trauma [1]. Cortisol is secreted in response to stimulation of the adrenal cortex, which occurs from increasing adrenocorticotrophic hormone (ACTH) levels after initiation of surgical procedures.

As a result of the physiological consequences of cardiopulmonary bypass (CPB), hormone secretion and blood glucose levels change and these alterations render the patients undergoing cardiac surgery vulnerable to morbidities including infection, atrial fibrillation, low cardiac output, prolonged respiratory depression, and cerebrovascular accident [2–4]. Recognition of the fact that anaesthetic agents have an important role in the stress response led to several studies that focused on inhibition and prevention of the stress response [5]. In studies of the effect of stress response inhibition on surgical outcomes, it was observed that the stress response can be changed with the use of different analgesic-anaesthetic techniques. It was demonstrated that anaesthetic agents had protective effects against ischaemia-reperfusion damage not only during the electrical phase but also during the inflammatory phase [6].

In this study, we compared the effects of propofol/desflurane anaesthesia in subanaesthetic doses and total intravenous anaesthesia (TIVA) on haemodynamics and stress responses in patients undergoing cardiac surgery.

## 2. Materials and Methods

**Figure 1:**
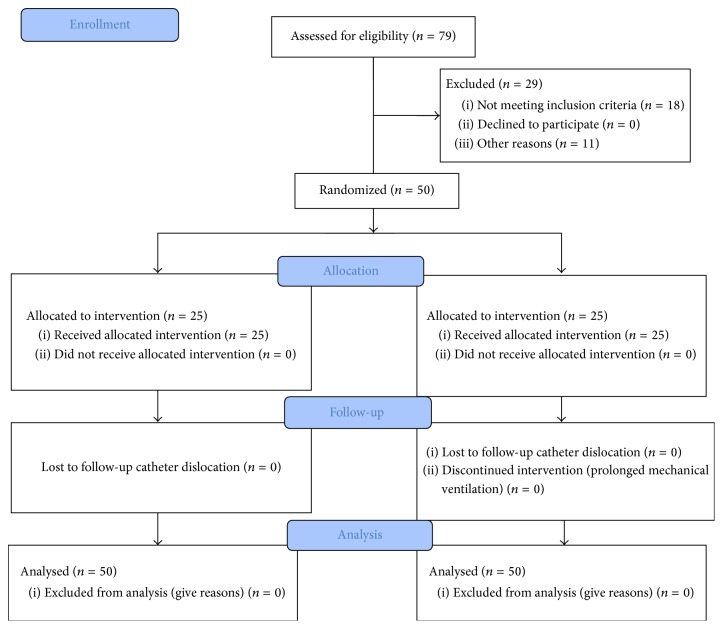
Flow chart of the study groups.

The study was approved by the ethics committee of Erzincan University (Ethics date and number: 28/01/2014, 1–5). All patients gave consent to take part in the research. From a total of 79 patients, 68 patients undergoing elective coronary artery bypass grafting (CABG) with cardiopulmonary bypass (CPB) were selected for the study. However, 18 patients excluded according to exclusion criteria (Figure 1). Therefore, a total of 50 patients were included in the study. A total of 50 patients, all over 30 years of age, with an American Association of Anesthesiologists (ASA) score of III and scheduled to undergo primary elective three-vessel coronary artery bypass grafting surgery were included. Patients were excluded from the study if they had suffered a myocardial infarction within the last month, had high myocardial ischaemic markers within the last 24 hours, received steroids over the long-term, had diabetes, had a low left ventricular ejection fraction (<50%), or had a high serum creatinine (>1.2 mg/dL). Patients having a clear indication for combined or emergency surgery were also excluded.

All patients were premedicated with 5 mg diazepam the night before as well as one hour before the operation. In the operating room, standard monitoring included electrocardiography, pulse oximetry, and invasive blood pressure measurement.

Anaesthesia was induced with 1.5–2.0 mg/kg propofol and 5–10 *μ*g/kg fentanyl. Rocuronium at a dose of 1 mg/kg was used for neuromuscular blockade. Patients were mechanically ventilated with the ventilator set as follows: volume control (VC) mode, air/oxygen mixture at 40/60%, respiratory rate at 12 L/min, positive end-expiratory pressure (PEEP) at 0 mbar, maximum pressure (*P*_max_) at 30 mbar, and tidal volume (TV) at 7–10 mL/kg. Central venous pressure was monitored with a 7 F central venous catheter (arrow sheath), which was introduced into the internal jugular vein.

Patients were randomly divided into two groups. In group PD (subanaesthetic dose of propofol-desflurane), anaesthesia was maintained with a propofol (2% propofol, Fresenius Kabi) infusion (2-3 mg/kg/h), fentanyl infusion (3–5 *μ*/kg/h), and desflurane (Suprane, Baxter, Puerto Rico, ABD) inhalation at a dose of 1 minimum alveolar concentration (MAC), whereas, in group P (TIVA group), anaesthesia was maintained with a propofol infusion (5-6 mg/kg/h) and fentanyl infusion (3–5 *μ*/kg/h). All medications were continued at the same doses during CPB.

Body temperature was monitored with a nasopharyngeal probe (Bicakcilar, Istanbul, Turkey).

End-tidal CO_2_ levels were measured using a Nihon Kohden Life Scope 14. Depth of anaesthesia was monitored using bispectral index (BIS) measurements (Aspect Medical Systems BIS VISTA*™*, Covidien) with the unconscious index being within surgical anaesthesia levels (40–60).

### 2.1. Sample Collection

Blood samples were drawn preoperatively (T0), after initiation of CPB but before cross-clamping the aorta (T1), after removal of the cross-clamp (T2), and at the 24th postoperative hour (T3).

### 2.2. Laboratory Assessment

Samples were centrifuged at 4000 rpm for 15 minutes using a NF 1200 R centrifuge system and stored at −80°C. At the time of laboratory assessment, the samples were taken out of the freezer and were allowed to thaw to room temperature. Serum insulin levels were detected using Immulite 2000 insulin kit, and brain natriuretic peptide (BNP) levels in heparinised plasma were detected using Immulite 2000 kit for Immulite 2000 XPi system (Siemens). Serum cortisol was detected using the Advia Centaur XP kit for the Advia Centaur XP System (Siemens). Serum creatinine kinase (CK) and creatinine kinase muscle-brain (CK-MB) were detected using Beckman Coulter AU2700 PLUS system, and serum C-reactive protein (CRP) was detected using Beckman Coulter Immage 800 system.

### 2.3. Statistical Analysis

All statistical analyses were performed using the Statistical Package for Social Sciences for Windows version 15.0 (SPSS Inc., Chicago, IL). Power analysis revealed that, to reach 90% statistical power, the sample size should total 50 for 95% confidence and 0.05 significance levels. Normal distribution of variables was tested using the Kolmogorov-Smirnov test. Continuous demographic variables were compared using the independent samples *t*-test. A paired samples *t*-test was used for repeated cortisol and insulin measurements. The Mann-Whitney *U* test was used for comparison of nonnormally distributed data. *p* value of less than 0.05 was considered statistically significant. For an effect size of 0.32 with a 95% confidence level and 0.05 significance, the power was calculated as 0.822 for a two-tailed *t*-test.

## 3. Results

The two groups were similar regarding baseline characteristics including age, body mass index, Euroscore, and preoperative ejection fraction (Table 1). There were no significant differences between the two groups regarding systolic, diastolic, or mean arterial pressure values recorded after induction of anaesthesia and after intubation of the patient.

Mean arterial pressures recorded before and after sternotomy were significantly lower in group P than in group PD (*p* = 0.01 and *p* = 0.001, resp.). The mean arterial pressures recorded after removal of the cross-clamp and after cessation of CPB were also significantly lower in group P than those in group PD (*p* = 0.022 and *p* = 0.015, resp.; see Table 2).

Nitroglycerin (NTG) was used to treat systemic hypertension in 18 patients (70%) in group PD. NTG concentration varied from 1.3 ± 0.3 to 2.5 ± 0.9 *μ*g/kg/min. In group P, patients did not require the use of NTG.

Heart rate recorded after removal of the cross-clamp and after cessation of CPB was significantly higher in group PD than in group P (*p* = 0.012 and *p* = 0.019, resp.; see Table 2).

Preoperative serum cortisol levels were similar between the two groups, whereas there were significant increases in intergroup measurements performed before cross-clamping the aorta, after removal of the cross-clamp, and at 24 hours postoperatively (*p* > 0.005). Cortisol levels were significantly higher in group PD compared to group P (*p* < 0.005) (Table 3).

Insulin levels were not significantly different between the two groups at all-time points, whereas, in both groups, T2 and T3 measurements revealed higher insulin levels when compared to T0 (baseline) measurements (*p* < 0.005). Among all biochemical parameters analysed, CK-MB was the only parameter that was significantly different between the two groups, and it was significantly lower in group P compared to group PD (Table 3).

## 4. Discussion

The stress response in cardiac surgery patients is related to increased secretion of stress hormones and cytokines. Cardiopulmonary bypass (CPB) induces a variety of mechanisms, such as decreased drug metabolism, damage to circulating blood cells, and adsorption of plasma proteins. As a result of the evoked immune response, the systemic inflammatory response is activated [7, 8]. When triggered as a response to surgery, the stress response is characterized by sympathetic activation, which is accompanied by an endocrine stress response, including increased pituitary gland secretion and insulin resistance.

Activation of the stress response induces tachycardia and increases myocardial oxygen demand which is accompanied by an increase in peripheral vascular resistance and oxygen consumption. Sympathetic stimulation, which is triggered via *α*-receptors, causes coronary vasoconstriction and further induces myocardial ischaemia [5].

Inhalation anaesthetics, in particular, were demonstrated to protect the myocardium against ischaemia by mimicking ischaemic preconditioning and diminishing the adverse effects of reperfusion injury [5, 9]. The effects of morphine and other opioids on the surgical stress response have been well documented in patients undergoing cardiac surgery [10].

In our study, patients receiving TIVA had better haemodynamic, metabolic, and hormonal stress markers when compared to those receiving subanaesthetic doses of propofol and desflurane, indicating that TIVA provided better prevention against the stress response in patients undergoing coronary artery bypass grafting (CABG) under CPB. Significant changes in the stress response were associated with improvements in clinical outcomes. Total intravenous anaesthesia can reduce the sympathetic activation of the vascular system, leading to decreased cardiac output, vasodilation, and hypotension. Besides the endocrine and metabolic changes, the effects of anaesthetics and analgesics on the surgical stress response have also gained importance [11].

Patients in the TIVA group (group P) were extubated, on average, 2.3 hours earlier, stayed for a shorter time in the intensive care unit, and were discharged earlier to home when compared to those in group PD. These differences can be attributed to the better haemodynamic status achieved in the TIVA group. Moreover, the occurrence rate of atrial and ventricular arrhythmias was also lower in group P than in group PD (*p* = 0.09).

During induction, propofol decreases the systolic and diastolic blood pressure by approximately 20–40% with minimal change in heart rate [12]. The initial propofol-mediated decrease in arterial blood pressure continues during anaesthesia without a simultaneous increase in heart rate and the pulse rate does not change [13]. In our study, the patients' central venous pressure ranged from 8 to 10 mmHg. In patients with systolic blood pressure below 90 mmHg, we started induction after volume replacement was achieved. Therefore, we believe that the haemodynamic changes in the TIVA group were due to propofol.

A rapid increase of desflurane concentration in humans increases sympathetic activity, hormonal variables, heart rate, and arterial blood pressure more than an equivalent increase in isoflurane concentration [14].

Schöffmann et al. [15] studied piglets receiving TIVA anaesthesia with propofol and fentanyl and found that mean arterial pressures did not significantly differ before and after surgery, whereas plasma cortisol levels were significantly lower after the operation when compared to those recorded before the operation. Ihn et al. [16] reported that patients receiving TIVA anaesthesia with propofol and remifentanil had a greater reduction in cortisol levels than those receiving sevoflurane anaesthesia. In our study, preoperative serum cortisol levels were similar between the two groups, and we observed a significant increase in serum cortisol levels at T1, T2, and T3 (*p* ≤ 0.05) in both groups. When we compared both groups, cortisol levels were significantly higher in group PD than group P (*p* ≤ 0.05).

Since increasing stress hormone levels are generally associated with an increase in patient morbidity, this issue has been subject of many studies for the purpose of diminishing stress hormone levels in patients undergoing cardiac surgery. Kostopanagiotou et al. [17] found that propofol did not differ from desflurane but caused a significantly lower stress response in terms of cortisol and ACTH levels when compared to sevoflurane. Burton et al. [18] demonstrated that the stress response during surgical trauma caused a change in hormone secretion and plasma cortisol levels and also inhibited release of insulin.

It was reported that a fivefold or more increase in CK-MB levels was associated with an increased risk of myocardial infarction and 30-day mortality after CABG. Moreover, high postoperative CK-MB levels were reported to be associated with 6-month and 1-year mortality [19]. In our study, CK-MB levels were significantly lower in all measurements in group P compared to group PD.

Brain natriuretic peptide is secreted from ventricular myocytes as a response to ventricular failure. Studies demonstrated that BNP levels are positively correlated with the degree of ventricular failure [20]. In our study, although both TIVA and subanaesthetic propofol combined with desflurane did not cause ventricular failure, the myocardium might have been exposed to transient ischaemia. Unlike BNP, CK-MB is released during myocardial ischaemia [21]. Moreover, BNP levels may not increase within the first 24 hours of myocardial failure. We think that this was why CK-MB levels, but not BNP levels, were significantly different between the two groups. An increase in CRP is likely to be more prominent secondary to surgical trauma versus secondary to the inflammatory response against ischaemia and stress during CABG. Therefore, we suspect that this is the main reason why we did not find any difference in CRP levels between groups.

An international consensus has been reached regarding the potential of volatile anaesthetics in decreasing perioperative and intensive care mortality rates in cardiac surgery patients. It was accepted that further study on this issue is warranted [22]. However, these studies lack data regarding patients undergoing coronary artery bypass and valvular surgery. In our study, blood cortisol levels were found to be significantly lower in the TIVA group than in the group given a subanaesthetic dose of propofol and desflurane.

Total intravenous anaesthesia with propofol and remifentanil was suggested to be a safe option in cardiac surgery even in patients with severe left ventricular dysfunction [23, 24]. In cardiac surgical patients, alleviation of the neuroendocrine stress response that may be evoked by intubation, sternotomy, and CPB is of crucial importance. In our study, cortisol release, which increased as a result of the stress response, was better inhibited by the use of TIVA when compared to a subanaesthetic dose of propofol and desflurane. Moreover, better inhibition of sympathetic stimulation provided better haemodynamic stability. However, patients receiving desflurane required higher doses of nitroglycerin to control hypertension.

Our study had several limitations. The major limitations were the inclusion of ASA III patients and the single institution setting. Most of our patients had diabetes, and therefore we could not include these patients in the study. On the other hand, some of our patients had low left ventricular ejection fractions or had suffered a myocardial infarction within the last month. All of our patients had three-vessel disease to keep equal cross-clamp times between patients. These limitations allowed us to include more patients.

In conclusion, cortisol levels were significantly lower before cross-clamping the aorta, after removal of the cross-clamp, and at the 24th postoperative hour in the present study. Furthermore, we found that CK-MB levels were significantly lower in all measurements. Since both parameters are related to postoperative myocardial function and mortality, TIVA anaesthesia resulted in lower blood pressure and CK-MB compared to a subanaesthetic dose of propofol with desflurane anaesthesia in patients undergoing CABG for three-vessel disease.

## Figures and Tables

**Table 1:** Comparisons of patient variables.

Parameters	Group PD mean ± SD	Group P mean ± SD	*p* value
Age (years)	65.600 ± 18.4	63.520 ± 17.9	0.420
Weight (kg)	73.160 ± 13.21	79.360 ± 8.31	0.087
Height (cm)	164.760 ± 18.67	167.600 ± 11.78	0.263
BMI (kg/m^2^)	26.508 ± 2.31	28.532 ± 3.42	0.147
Euroscore	2.316 ± 0.56	2.660 ± 0.45	0.324
ASA	2.800 ± 0.12	2.920 ± 0.11	0.230
EF	55.240 ± 3.67	54.120 ± 3.12	0.589

SD: standard deviation; EF: ejection fraction.

ASA: American Association of Anesthesiologists; BMI: body mass index.

**Table 2:** Comparison of haemodynamic data between two groups.

Parameters	Group PD mean ± SD	Group P mean ± SD	*p* value
Heart rate (beats/min)			
Baseline	72 ± 10.11	69 ± 9.9	0.782
After induction	76 ± 9.5	78 ± 6.3	0.506
Before clamping	63 ± 6.5	67 ± 3.1	0.604
After declamping	92 ± 3.4	81 ± 4.7	0.013
Postoperative	81 ± 4.5	84 ± 5.3	0.302

Mean arterial pressure (mmHg)			
Baseline	85.50 ± 11.22	86.50 ± 9.34	0.630
After induction	80.50 ± 9.63	78.50 ± 10.30	0.550
Before clamping	86.00 ± 8.75	74.00 ± 11.63	0.032
After declamping	85.60 ± 10.32	78.80 ± 11.21	0.021
Postoperative	87.99 ± 9.64	81.60 ± 8.71	0.019

**Table 3:** Serum cortisol and CK-MB levels between groups.

Time period	Group P mean ± SD	Group PD mean ± SD	*p* value
Serum cortisol levels (mg/dL)			
Preoperative	19.52 ± 9.24	20.48 ± 8.81	<0.001
Before clamping	27.07 ± 12.37	41.09 ± 15.37	<0.001
After declamping	46.22 ± 17.66	60.49 ± 9.76	<0.001
Postoperative	20.54 ± 6.94	32.13 ± 21.53	<0.001

Serum CK-MB levels (IU/L)			
Preoperative	10.40 ± 3.990	14.84 ± 6.712	<0.05
Before clamping	20.80 ± 9.849	31.36 ± 19.579	<0.05
After declamping	32.16 ± 11.761	44.38 ± 21.346	<0.05
Postoperative	39.12 ± 30.158	65.20 ± 37.621	<0.05
